# Photoprotective Effect of *Annona muricata* L. Extracts in Rats Exposed to Ultraviolet Radiation via P53 and RB Gene Expression

**DOI:** 10.3390/molecules30173518

**Published:** 2025-08-28

**Authors:** Juan Carlos Pizano-Andrade, Belinda Vargas-Guerrero, Jesus Vargas-Radillo, José Alfredo Domínguez-Rosales, Efigenia Montalvo-González, Ramon Rodriguez-Macias, Pedro Macedonio Garcia-López, Margarita del Rocio Romero-Verdín, Mario Alberto Ruiz López

**Affiliations:** 1 Botany and Zoology Department, CUCBA, University of Guadalajara, Zapopan 44600, Jalisco, Mexico; pizanjc@gmail.com (J.C.P.-A.); ramonrod@cucba.udg.mx (R.R.-M.); macedonio.garcia@academicos.udg.mx (P.M.G.-L.); margarita.rverdin@academicos.udg.mx (M.d.R.R.-V.); 2 Department of Molecular Biology and Genomics, University Center for Health Sciences, University of Guadalajara, Guadalajara 44340, Jalisco, Mexico; bevargro@hotmail.com (B.V.-G.); dominique14@yahoo.com (J.A.D.-R.); 3 Department of Wood, Pulp and Paper, University Center for Exact Sciences and Engineering, University of Guadalajara, Guadalajara 44430, Jalisco, Mexico; j.vargas@academicos.udg.mx; 4 Integral Food Research Laboratory, National Technological of Mexico/Technological Institute of Tepic, Av., Tepic 63175, Nayarit, Mexico; emontalvo@ittepic.edu.mx

**Keywords:** acetogenins, graviola, skin tumors, phenols

## Abstract

A current problem is the increase in skin damage, including cancer, caused mainly by prolonged exposure to ultraviolet rays from sunlight. Therefore, the aim of this work was to study the photoprotective effect to ultraviolet radiation of phenolics and acetogenic-rich extracts obtained from *Annona muricata* leaves applied to the skin of rats by means of gene expression in *P53* and *Rb*, involved in tumor processes due to cell damage, in addition to the content of phenols, acetogenins and antioxidant activity present in the extract, which presented a total phenol content of 61.5 mg EAG/100 g of dry sample and flavonoids of 50 mg EQ/100 g. HPLC analysis revealed that the major compound was shikimic acid, followed by gallocatechin and 13 other phenols. DPPH analysis showed an inhibition of 64.37% and FRAP showed a value of 28,880 µmol Eq trolox/mL. The presence of acetogenins was verified by Kedde’s reagent in HPTLC. Histopathological findings in the treated groups (T4, T5) suggest thickening of the epidermis, which could be due to fibroblast proliferation. The results show a higher increase in *P53* and *Rb* gene expression with the tested extracts compared to the positive control group, so it can be concluded that the extracts have positive effects.

## 1. Introduction

The skin, as the largest protective part of the body, is constantly is exposed to sunlight, making it critical to protect this organ from its harmful effects. When the skin is subjected to long-term exposure to UV radiation, biological responses are induced, such as the development of erythema, edema, cell burns, hyperplasia, immunosuppression, DNA damage, photo-ageing, change in gene expression and non-melanoma skin cancer, which is the most common type of cancer in the world [1,2,3].

Skin tumors are currently one of the most severe effects of ultraviolet radiation, and this neoplasm is becoming more and more frequent, with melanoma-type cancer increasing by 40% and non-melanoma cancer by up to 20% [4].

Genes involved in tumor development have been reported, including *P53* and *Rb*, both of which are involved in cell cycle signaling. When *P53* and *Rb* gene expression decreases, the chances of cumulative cell damage increase, which can ultimately trigger tumorigenesis, attributable to the UV component of sunlight [2].

The study of secondary metabolites from plants to prevent and treat tumors and skin damage has been of growing interest for some time. Soursop (*Annona muricata*), a popularly edible plant in many countries of the Americas, has been found to be compounds of great medical interest for their pharmacological effects; several parts of this plant are used traditionally, as leaves, seeds, bark, fruit, stem, roots and twigs, and a range of different methods for preparation is reported, such as infusions, pastes and decoctions [4].

Several studies have shown that *A. muricata* has different properties, such as anticancer, antiulcer, antidiabetic, antiprotozoal, antidiarrhea, antibacterial, antiviral, antihypertensive, and wound healing [5,6,7].

The leaves and fruits to has been tested in vitro in colon cancer cells (HT-29, HCT-116), pancreatic carcinoma (PACA-2) and colon adenocarcinoma (HT-29) cell lung tumor cell lines (A-549) and prostate cancer (PC-3) cells [7,8]; however, no studies have been reported in skin cancer with extracts of *A. muricata*, of in vivo models; only studies with non-melanoma skin cancer cells are reported [2].

The main active components of *A. muricata* are acetogenin (annomuricins and annonacin), alkaloids, and flavonoids (quercetin) [9]. These compounds are responsible for the biological activity of *A. muricata*. Acetogenins have been reported to have antitumor activity and polyphenols have been reported to have photoprotective activity [10,11,12] However, more studies are required to know this important antitumor and photoprotective property, such as the effect of these compounds on gene expression *in vivo* models exposed to ultraviolet radiation.

Therefore, this study evaluated the photoprotective effect of phenolic and acetogenins extracts from *Annona muricata* leaves in a murine model exposed to UVR, as well as the expression of the P53 and RB genes. This research is being conducted for the first time in an in vivo, histological, and molecular study.

## 2. Results

### 2.1. Phenolic Compounds and Acetogenins

The results of the methanolic extraction revealed a yield of 35% (dry solids), while the petroleum ether extracts were greater than 38% (dry solids).

The quantification of total phenolic, flavonoids and antioxidant capacity of leaf extract are shown in Table 1, with values of 61.5 mg GAE/100 g of total phenolics and 50.3 mg QE/100 g of flavonoids. While the results of the antioxidant capacity with DPPH showed that the leaf extracts had an inhibition percentage of 64.37%, the extract with FRAP presented an antioxidant capacity of 28.280 mmol Eq trolox/mL.

### 2.2. Sun Protection Factor (SPF)

The evaluation of the sun protection factor obtained from the extracts of purified phenolic compounds in leaf was 18.9 SPF, and that from the acetogenin extract was 26.2.

### 2.3. HPLC Characterization of Phenolic Compounds

The results of the characterization of the purified leaf extract can be seen in Table 2. It can be noticed that the major compound is shikimic acid with 612 mg/g of dry sample, while the minor compound was protocatechuic acid with 0.0305 mg/g of dry sample; 15 different phenolic compounds were identified, and, in total, 642.64 mg of phenolic compounds were extracted per gram of dry sample.

The results of thin layer chromatography (TLC) showed the presence of acetogenins in the ethereo extract, and three distinct bands were observed, suggesting the presence of three fractions of acetogenins.

### 2.4. Histologic Analysis

Macroscopically, no skin damage of any kind was observed in the T1 group during the experimental period. In contrast, the rats in group T2 from the first session presented diffuse erythema on the skin, which disappeared or diminished in the first hours after exposure to radiation. With time, the erythema became permanent and was accompanied by a geometric pattern that became larger. Microscopically there was evidence of inflammation, thickening of the epidermis, presence of fibroblasts, lymphocytes, edema, and collagen disorganization.

Macroscopically, in groups T3, T4 and T5, no reddening of the skin was observed. Microscopically, only in group T2 were signs of inflammation observed, as well as a statistically significant thickening of the epidermis of 61.58 ± 27.26 (*p* < 0.05). There was no significant statistical difference between groups T2 and T3, and neither between groups T3 and T4; however, group T1 presents less thickening compared against groups T2, T3, T4 and T5. In the fibroblasts, groups T2 (2.7 ± 0.67 mm^2^), T4 (2.8 ± 0.86) and T5 (2.566 ± 0.83 mm^2^) are statistically similar, and groups T3 and T1 (2.061 ± 0.80 and 1.577 ± 0.64, respectively) are different from those already mentioned and different from each other (Table 3).

### 2.5. P53 and RB Gene Expression

**Figure 1:**
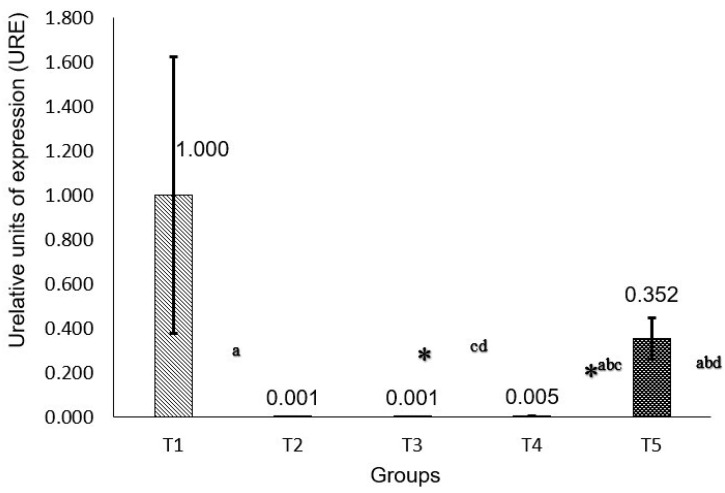
Gene expression of P53. Calculation 2^−ΔΔCT^. * *p* < 0.05 comparing against group T1: (a) *p* < 0.05 group T3 against T2; (b) *p* < 0.05 group T3 against T5; (c) *p* < 0.05 group T3 against T4; and (d) *p* < 0.05 group T5 against T3. * Statistically significant.

For the analysis of P53 gene expression (Figure 1), it is observed that the T1 group is statistically significant when compared to all others. The T3 and T4 groups show low expression, similar to the T1 group. As for the T5 group, it shows a higher expression than the T3 and T4 groups.

**Figure 2:**
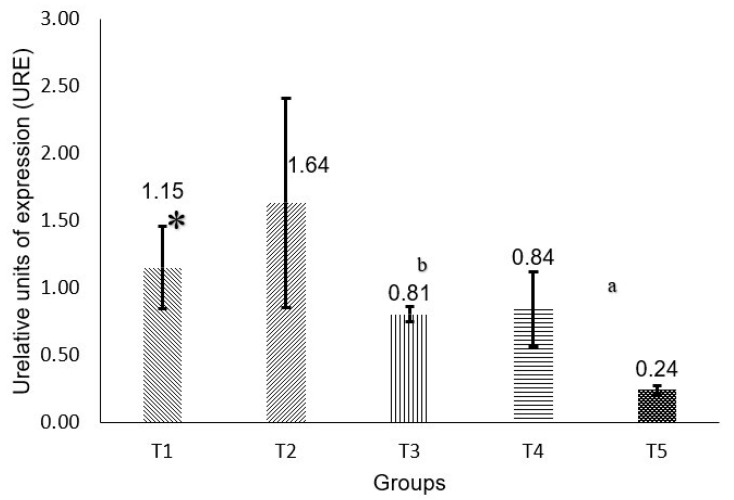
Gene expression of RB Calculation by 2^−ΔΔCT^. * *p* < 0.05 comparing against group T1: (a) *p* < 0.05 group T3 against T5, (b) *p* < 0.05 group T3 against T5.

Regarding *RB* gene expression (Figure 2), it can be observed that there is a significant statistical difference when comparing the T5, T4, and T3 groups.

## 3. Discussion

The protective effect of natural products, such as photopreventive effects against skin damage, have been reported, as they are able to absorb UV rays and act as filters to prevent damage to DNA, to the skin; some of these metabolites are phenolic compounds [10].

The analysis of the content of phenolic compounds and total flavonoids, families that contain molecules of interest with photoprotective properties, is the first step to characterize these products of natural origin. In this sense, Ref. [10] lower values in soursop leaf extracts reported ~42 mg/g from a methanolic extract, while the results of the present work, show that the methanolic extract showed 61.5 µg GAE/100 g of dry leaf; this difference could be explained by different location and origin plant material.

Antioxidant activity shows DPPH 64.37% and FRAP 28.8 mmol eq trolox/mL, similar to other studies [13].

In relation to the sun protection factor (SPF), there are several studies in which the SPF of compounds of different natures are analyzed; for example, oils, such as coconut oil and olive oil, which report SPF values of 7.1 and 7.5, respectively [14]. The above difference may be due to the fact that the oils contain mostly essential oils instead of phenolic compounds, and they have a greater capacity to absorb light, which makes the extracts used in this study potential components for use in formulations to combat the damage generated by UV light. Likewise, the SPF of aqueous extracts of *Citrus unshiu* peel has been reported [14,15] with a value of 6.8 to 16.88, as well as extracts of aerial parts of *Capnophyllum peregrinum* (L.), with an SPF of 35.1. Likewise, natural extract has the advantage over commercial sunscreens that do not present active compounds (UV filters) that are discarded into the environment, acting as persistent pollutants and accumulate in the fatty tissues and body fluids of living organisms. However, it would be interesting to conduct further research on the existence of any toxic products in theses extracts due to the effect of ultraviolet light.

The generation of reactive oxygen species is known to trigger oxidative stress that subsequently alters different metabolic processes, leading to cell death through abnormal biochemical and/or physiological processes. The identification and quantification of antioxidant molecules from natural products is an area of great interest. In one study [16] they evaluated antioxidant activity of extracts obtained from *A. squamosa* and *A. reticulata* showing better results than *Annona muricata*. In another study [17], evaluating the antioxidant effect of methanolic extracts from *A. muricata* leaf, an inhibition percentage of 87% was shown, higher than the 64.37% inhibition of the purified methanolic extract reported in the present study with the DPPH assay.

In relation to the HPLC characterization, 15 different phenolic compounds present in the *A. muricata* leaf could be identified. This contrasts with the findings of Ferreira et al. (2013) [17] which shows a chromatographic profile of an aqueous extract of *Annona muricata* leaves, with only 7 phenolic compounds (not mentioned), while Jiménez et al. (2014) [18] identified 16 compounds in a HPLC analysis with mass detector, which is quite close to what was found in the present project. Although the compounds found were different, this suggests that the extraction process performed, the configuration of the equipment, the sensitivity or the amount of standards used as well as collection times and locations influence the identification of compounds, make a difference.

In the present work, the extraction was carried out with the support of ultrasound or ultrasound-assisted extraction (UAE), in contrast to what was reported by other authors [3,18], who performed direct extraction with agitation and centrifugation. Another soursop leaf characterization [19] reports the presence of 24 phenolic compounds, where the first 15 are similar to what Nolasco-González et al. (2022) [10] found. In addition, the majority was epigallocatechin, while, in this report, the majority was shikimic acid. In relation to this, it should be noted that the extraction procedure was different, since acetone was used as solvent, in addition to the fact that the amplitude, pulse cycle and time were controlled, i.e., different ultrasound conditions. On the other hand, the extract was subjected to the process to remove chlorophylls; all of the above could have contributed to the difference in the compounds found. Different authors mention that the phenolic compounds obtained may vary, mainly due to the extraction method, processing or mechanism of detection and identification by other analytical procedures, as Nawwar et al. (2012) [20] performed with fractional extractions of soursop leaf using as solvent a hydroalcoholic mixture of EtOH-H2O (3: 1) with reflux for 8 h; subsequently seven fractions were passed by column, and the identification was performed by 2-dimensional chromatography and nuclear magnetic resonance, finding quercetin, gallic acid, epicatechin, catechin, chlorogenic acid, and kaempferol, being similar in our work both Catechin and chlorogenic acid.

On the other hand, genes such as *p53* and *Rb*, which usually remain inactive or very little active, are activated when there is stress or damage affecting the cells. Although an increase was expected in the T2 group, a decrease was observed; in this sense, there are reports [1] in which *p53* is inactivated after intense radiation. Furthermore, solar exposure can generate a mutation in the gene, so when designing the primers for the wild-type gene, it is possible not to detect the expression of the mutant gene [19].

The *RB* activation pathway can occur prior to activation of the *p53* gene; however, the decreased expression of *p53* suggests that it is being activated independently. One possibility is that the N-terminal region of *RB* is being phosphorylated, which causes cell cycle arrest and increases cell survival in stress processes [21], which could explain the results obtained in *RB* expression.

As for *p53* gene expression levels, in the T5 group there is a decrease compared to T1. It does not significantly resemble the T2 group. This suggests that the emission of UV rays was adsorbed to a large extent by the extract even though it could have protected as reported in some studies [13]. Other studies presented similar results; in one study [22] a green tea extract was evaluated in human skin irradiated with UVB light; the results obtained were the reduction of *p53* expression. It should be noted that the phenolic compounds present in green tea are catechin, epicatechin, epigallocatechin, epicatechin gallate, which are similar to some of those found in the analysis of the *A. muricata* extract used (in gallocatechin, catechin and protocatechuic acid) [23].

The exposure of the different groups to UV light generated different responses when exposed to these emissions; it should be noted that there are reports that mention different doses of exposure, some that can be considered toxic and some that are considered non-toxic, in which it seems to induce a stabilization of *p53*, mainly in the UVA and UVB lengths [24].

The results showed thickening of the epidermis in the T2 and T3 groups, which is suggestive of damage to the epidermis due to exposure to radiation. This has been reported in previous works [2] in which mice were exposed to ultraviolet radiation with damage similar to those found here, such as inflammation, edema, vessel constriction, and collagen disorganization, among others. The differences were the models used because in their study dimethyl benzaanthracene-tetradecanoylphorbol-13-acetate as a promoter of tumor damage, in contrast to our work where the model used was 20 days, 1.5 h per day of exposure to UV light, which confirms that it is feasible to observe and study the initial damage produced by UV light.

Similar studies [25], using an extract of *Morinda citrifolia* (Rubiaceace) report a decrease in the inflammatory process, as well as a less expressed hyperemia (increase of blood in an organ or tissue) and a lesser affectation in the thickening of the epidermis compared to the untreated groups, where inflammation was mostly observed. The above changes were also observed in our research work, as the greatest damage was observed in the T2 and T3 groups. The treated groups showed a decrease in damage characterized by reduced epidermal thickness and less inflammation, in addition to a decrease in fibroblasts and preserved collagen.

The differences between these studies are the presence of quercetin-3-O-rutinoside, rutin and kaempferol compounds in the extracts of *M. citrifolia* (mountain soursop), while in the extracts of *A. muricata* (soursop) obtained here, up to 15 different compounds were identified. Another difference was that three different concentrations of *M. citrifolia* were evaluated, which were 0%, 10% and 15%, while the soursop extracts were used directly. The lamps used were similar to those used in this project in terms of the irradiation spectrum, since they used a Repti glo 10.0 lamp (Exo-Terra). On the other hand, the irradiation protocol was shorter since it was only 7 days for 2 h per day at a distance of 20 cm from the back of the skin [26].

Fibroblasts are a type of cell that contributes to the formation of connective tissue, and the secretion of collagen (which is a structural protein of tissues), as well as being involved in wound healing [27]. Considering that the thickness of the epidermis (as an indicator of damage) decreases in the T3 and T5 groups, added to the increase in the fibroblast count in the same groups, this suggests that the compounds tested fulfill a beneficial function in the functions of fibroblasts to form connective tissue. In this regard, Czemplik et al. (2017) [26] report that the use of an extract of phenolic compounds from flaxseed caused increased fibroblast growth and viability by 20%, where the main component reported was 4-hydroxybenzoic acid, which is one of the minor compounds present in the *A. muricata* leaf extracts used. It has been described that exposure to UV light disrupts the function of fibroblasts by generating reactive oxygen species, so an extract of phenolic compounds such as the one used can protect against fibroblast damage in the skin [28,29].

The T4 group was one of the groups with less epidermal thickening and a lower fibroblast count per mm^2^, which stands out because according to what was reported by Coria-Téllez et al. (2018) [30] and Jacobo-Herrera et al. (2019) [19] acetogenins from various types of extracts (leaf, pulp), have been used in various skin alterations, including different types of skin cancer. On the other hand, Hamizah et al. (2012) [29] mention that ethanolic extracts were able to generate 0% incidence of initiation and promotion of tumors in mouse skin, in an induction model with 7,12-dimethylbenzanthracene.

In other studies, Refs. [23,31], it was found that the use of green tea, which is rich in flavonoid-like compounds such as catechin, epicatechin, epigallocatechin, and epicatechin gallate, in clinical studies showed a decrease of up to 38.9% in the amount and intensity of observed sunburn. It should be noted that in the characterization studies of soursop phenols presented in this report, gallocatechin, catechin, and protocatechuic acid are found, which could suggest that protection could be comparable to that of green tea. Another study [32] analyzed the role of flavonoids in photoprotection; among the compounds mentioned and analyzed are caffeic acid and p-coumaric acid, which are present in soursop. Similarly, Korać and Khambholja, (2011) [33] mentioned that phenolic acids have an important use in skin protection against ultraviolet radiation, which denotes the relevance of the phenolic acids found in the present work, which were protocatechuic acid, 4-hydroxybenzoic acid, chlorogenic acid, 4-hydroxyphenylacetic acid, vanillic acid, syringic acid, 3-hydroxybenzoic acid, caffeic acid, p-coumaric acid, trans-ferulic acid, ellagic acid and the most important, which was shikimic acid.

As mentioned above, exposure to UV light causes considerable damage to the skin at different levels, in addition to inducing the generation of ROS. The use of antioxidant compounds capable of inactivating ROS is a current approach to prevent skin damage caused by sun exposure, as it has been observed that sunscreens are formulated based on protection against erythema and not against free radical generation or damage, suggesting that prolonged sun damage would increase with UV exposure [28].

## 4. Materials and Methods

### 4.1. Plant Material

The samples were obtained in the municipality of Tecoman, Colima, Mexico. The plant material was botanically identified by the National Laboratory of Plant Identification and Characterization (LANIVEG in Spanish) and deposited in the IBUG herbarium under voucher number 210429. The leaves were manually separated, ground, dried in an oven at 40 °C for 48 h and stored until use.

### 4.2. Extraction of Phenolic Compounds from Annona Muricata Leaves

The extraction of phenolic compounds from leaves was performed, using methanol as solvent, with slight modifications to remove chlorophyll as an impurity, to a volume of leaf extract, two volumes of chloroform, stirring for 5 min at room temperature, forming two phases, of which the lower phase corresponding to chloroform with chlorophyll was discarded. Later, two volumes of ethyl acetate were added to the purified fraction and homogenized by means of agitation for 1 min. Finally, it was filtered and concentrated in a rotary evaporator and preserved at 4 °C until use [34,35].

### 4.3. Phenolic Compounds Quantification

The Folin–Ciocalteu (FC) technique was applied to 1 mL of extract; 1 mL of FC reagent was added, shaken using a vortex, and 10 mL of 7% sodium carbonate (Na_2_CO_3_) and 25 mL of distilled water were added and mixed. It was left to stand for 90 min in total darkness, after which the absorbance was measured at 750 nm in a UV spectrophotometer. The content of phenolic compounds was calculated with a calibration curve of gallic acid as a standard constructed with a concentration (n = 3) from 0, 200, 400, 600, 800, 1000 y 1200 mg/L [36]. R^2^ = 0.964 was obtained.

### 4.4. Flavonoid Quantification

This was performed using the aluminum chloride (AlCl_3_) technique. To 1 mL of the sample (leaf extract), 0.3 mL of 5% NaNO_2_ was added, shaken for 5 min after which 0.3 mL of 10% AlCl_3_ was added and shaken again for five minutes, then 2 mL of 1 M sodium hydroxide (NaOH) and 10 mL of H_2_O were added, and shaking was done. Absorbance was measured in a spectrophotometer at 510 nm, and total flavonoids were calculated with quercetin equivalents with a calibration curve of 0, 200, 400, 600, 800, 1000 y 1200 mg/L in triplicate (n = 3) [36], with an R^2^ = 0.99.

### 4.5. In Vitro Sun Protection Factor (SPF) Evaluation

In the purified extract, a sample was taken to read the absorbance in the form of scanning in a spectrophotometer at 290 nm with an increment of 5 nm until reaching 320 nm, with measurements made in triplicate. The following formula was used for the sun protection factor [14]:

$$SPf\;spectrophotometric=CF\sum_{290}^{320}EE\left(\lambda \right)\times I\left(\lambda \right)Abs(\lambda )$$

where

CF is the correction factor,

EE(λ) is the erythemogenic effect of the wavelength,

I(λ) is the intensity of the sun at the wavelength, and

Abs(λ) is the observance at which it is being measured.

EE(λ) × I(λ) are the determined constants.

### 4.6. Antioxidant Capacity Assay

DPPH

A 1,1-diphenyl-2-picrylhydrazyl (DPPH) was used. The sample (30 μL) was reacted with 200 μL of DPPH solution (190 μM) using methanol as solvent. The absorbance was measured at 517 nm after 10 min using a 96-well multimode microplate reader (Synergy HT, Bio-Tek, Instrument Inc., Winooski, Vermont, USA) [37]. The results were expressed as a percentage by applying the following formula:

Radical scavenging capacity (%)=[(AnoSample−Asample)/AnoSample]×100

A Sample = absorbance of the DPPH mixed with the extracts, AnoSample = absorbance of the DPPH where the sample was replaced with methanol.

FRAP

A 10:1:1 (*v*/*v*/*v*) solution of sodium acetate buffer (0.3 M, pH 3.6), 10 mM TPTZ (2,4,6-tripyridyl-s-triazine) and 20 mM iron chloride hexahydrate was prepared. The solution was brought to 37 °C before mixing with the samples. Twenty-four μL of the extract was taken and added to the microplate, then mixed with 180 μL of FRAP solution using a multichannel dispenser and the absorbance was read at 595 nm after 30 min. A multimode microplate reader (Synergy HT, Bio-Tek, Instrument Inc.) was used. A calibration curve was performed with trolox (6-hydroxy-2,5,7,8-tetramethylchroman-2-carboxylic acid), an analog of vitamin E, against which the absorbances of the soursop extracts were calculated, so the results were expressed as mmol trolox equivalent per g dry weight (mmol TE/g DW) [37].

### 4.7. Characterization of Phenolic Extract by HPLC

Twenty μL of methanolic extract was injected after filtration with 0.45 μm diameter millipore filters into an HPLC system (Agilent Technologies 1260 Infinity, Waldbronn, Germany) equipped with a photodiode array detector and a C18 Reversed Phase HPLC Column (5 μm particle size, 4.6 mm diameter, 250 mm long; Thermo Fisher Scientific, Sunnyvale, CA, USA). The mobile phases consisted of 2% acetic acid-acidified water (eluent A) and acidified water (0.5% acetic acid)-methanol (10:90, eluent B). Phenolic compounds were identified in the wavelength range 280–320 and their quantification was performed with standard curves of various phenolic standards [38].

### 4.8. Extraction and Identification of Acetogenins

For the extraction, 0.5 g of ground dried leaves were used using 50 mL of petroleum ether as solvent, left to macerate for 3 days without agitation, at room temperature. Subsequently, it was filtered and a rotary evaporator was used for its concentration; it was kept at 4 °C until use [38].

The identification of the presence of acetogenins was performed by high performance thin layer chromatography (TLC). Glass plates of 10 cm by 10 cm of silica gel 60F254 were used; the mobile phase consisted of chloroform/methanol 9:1 (*v*:*v*) and Kedde’s reagent, which is specific for acetogenins [39].

### 4.9. Animal Model

Twenty-five male rats of the Wistar strain provided by the *Centro Universitario de Ciencias Biológicas y Agropecuarias* (CUCBA) biotherium were used. The rats were maintained in biotherium conditions with commercial food and water ad libitum. The conditions were light/dark cycles 12 × 12 h; relative humidity 50–55% at a temperature between 22 and 24 °C; the procedures were in accordance with NOM-062-200-1999 and international guidelines, and the handling of the rats was approved by CUCBA bioethics committee (CINV-C/034/2025, August 13 2025).

The animals were divided into 5 groups of 5 animals as follows:

- T1, not exposed to UV light and without treatment.
- T2, exposed to UV light and untreated.
- T3, treated with a commercial sunscreen with SPF 50.
- T4, treated with acetogenins extract.
- T5, treated with phenolic compound extract.

### 4.10. UV Light Irradiation Model

For irradiation of the rats, two ExoTerra 26 Watt UVB 150 lamps were used, with radiation similar to that of a desert climate (3650 Lux) (Mansfield, MA, USA) (manufacturer’s data).

The rats were placed in PVC traps with individual separators. The rats were dorsally depilated, then the treatments were topically administered and after 1 h, they were placed under the UV lamp at 10 cm. The UV exposure was performed for 90 min daily for 20 days [40].

### 4.11. Histologic Examination

At the end of the irradiation period, the sacrifice and necropsy protocol were performed on all the animals. A region of the dorsal skin was excised. Tissue samples were treated in 10% formalin for 48 h, then fixed in paraffin, and 5-micrometer sections were made and stained with hematoxylin-eosin [41] to measure thickening of the epidermis and presence of fibroblasts.

For RNA extraction from skin, 50 mg of tissue was taken and 1 mL of QIAzol (QIAGEN) was added to it. The tissue was then macerated in a homogenizer (Benchmark Microtube Homogenizer) using microtubes and 3.0 mm zirconium beads (Benchmark Scientific) (Sayreville NJ, USA).

### 4.12. Gene Expression Quantification

RNA extraction was performed using the RNeasy Protect Mini kit (QUIAGEN, Madrid, Spain) according to the supplier’s instructions. Subsequently, reverse transcription was performed from 2 µg of mRNA using the Transcriptor First Strand cDNA Synthesis Kit (Roche, Basilea, Switzerland) to synthesize complementary DNA. Subsequently, *P53* and *RB* gene expression was quantified by real-time PCR using the LightCycler^®^ FastStart DNA Master Plus SYBR Green I Kit (Roche, Basilea, Switzerland) and GAPDH (glyceraldehyde-3-phosphate dehydrogenase) was used as a reference gene.

For the design of the primers, as well as their analysis, the PrimerBlast online program from the NCBI website was used, as well as the specialized Oligo 6 software (Table 4). All samples were performed in triplicate. The relative expression was calculated through the 2^−ΔΔCT^ method.

### 4.13. Statistical Analysis

For the analysis of gene expression, an analysis of variance (ANOVA) and a post-hoc Mann–Whitney U test was performed using IBM SPSS version 27. Likewise, the analysis of variance (ANOVA) was applied in the histological analysis. For significant differences between groups, the Games-Howell test was applied with the Minitab^®^ 21.1.1 program. Values of *p* < 0.05 are considered significant.

## 5. Conclusions

Our results showed high polyphenol and flavonoid content in *Annona muricata* leaves, at 61.5 and 50.3 mg/g of sample, respectively, with shikimic acid as the main phenolic compound (612.53 mg/g). In the present study, gene expression of two tumor suppressor genes (P53 and Rb) was observed to increase in groups of rats treated with acetogenin and polyphenol extracts from *Annona muricata*. In terms of histological damage, no skin redness was observed in the groups treated with acetogenin and phenol extracts and with commercial sunscreen. Likewise, less thickening of the epidermis and collagen fibers was observed with the extracts rich in phenols and acetogenins, similar to the commercial sunscreen, indicating photoprotection in the rats treated with these extracts. This would indicate that the extracts used have a beneficial effect. This type of work is important for understanding the genetic mechanism behind the induction of skin cancer, as well as for researching other sources of natural compounds that can induce a photoprotective effect against the sun’s ultraviolet light.

## Figures and Tables

**Table 1:** Phenol and flavonoid content and antioxidant capacity (DPPH, FRAP) in methanolic extract *Annona muricata* leaves.

	Total Phenolic mg GAE/100 g	Total Flavonoids mg QE/100 g	DPPH % Inhibition	FRAP mmol Eq Trolox/mL
Leaf	61.5 ± 2.26	50.3 ± 3	64.37 ± 1.19	28.88 ± 0.278

**Table 2:** Phenolic compounds identified in *Annona muricata* leaves by HPLC.

	Phenolic Compound	Wavelength	ATR	Concentration (mg/mL)	mg/g Sample
**1**	Shikimic Ac.	270	2.429	12.251	612.533
**2**	Protocatechuic Ac.	270	15.671	0.006	0.305
**3**	Gallocatechin	270	17.573	0.159	7.970
**4**	4-Hydroxybenzoyl Ac.	270	18.6805	0.008	0.425
**5**	Chlorogenic Ac.	320	19.5575	0.017	0.858
**6**	4-Hydroxyphenylacetic Ac.	280	19.6455	0.103	5.141
**7**	Vanillic Ac.	270	20.2105	0.011	0.571
**8**	Siryngic Ac.	280	20.9	0.010	0.508
**9**	3-Hydroxybenzoic Ac.	300	20.6245	0.037	1.867
**10**	Caffeic Ac.	320	21.0605	0.011	0.536
**11**	Catechin	280	21.386	0.039	1.925
**12**	Rutin	270	23.3335	0.062	3.089
**13**	p-Coumaric Ac.	310	23.405	0.047	2.343
**14**	Trans-ferulic Ac.	320	24.353	0.055	2.753
**15**	Ellagic Ac.	270	24.9165	0.037	1.828
				Total	642.649

**Table 3:** Protective effect of phenol and acetogenin extracts from UV light on the epidermis and fibroblasts in rat skin (Media ± SD).

Groups	N	Media ± SD (µm)	Media ± SD (mm^2^)
T2	45	61.58 ± 27.26 ^a^	2.689 ± 0.67 ^a^
T3	45	49.66 ±15.82 ^ab^	2.061 ± 0.80 ^b^
T4	45	43.02 ±12.43 ^b^	2.80 ± 0.86 ^a^
T5	45	28.56 ± 7.91^c^	2.566 ± 0.83 ^a^
T1	45	18.66 ± 8.87 ^d^	1.5770 ± 0.64 ^c^

Means that do not share a letter are significantly different (*p* > 0.05). literal together, there is no significant difference.

**Table 4:** Primer sequence.

Gene	Sense	Anti-Sense
RB	CCAGCGGAGTCCAAATTCCA	TCCCGAGGGTCTACAGTGTT
P53	CCCCTGAAGACTGGATAACTGT	AGTTCCAGGTTCCTGTGCTG
GAPDH	AGACAGCCGCATCTTCTTGT	TACGGCCAAATCCGTTCACA

## Data Availability

Data are available on request from the corresponding author.
